# Aberrant Functional Connectivity of the Salience Network in Adult Patients with Tic Disorders: A Resting-State fMRI Study

**DOI:** 10.1523/ENEURO.0223-23.2024

**Published:** 2024-06-03

**Authors:** Linda Orth, Johanna Meeh, Delia Leiding, Ute Habel, Irene Neuner, Pegah Sarkheil

**Affiliations:** ^1^Department of Psychiatry, Psychotherapy and Psychosomatics, RWTH Aachen University, 52074 Aachen, Germany; ^2^Department of Psychiatry and Psychotherapy, University of Münster, 48149 Münster, Germany; ^3^Institute of Neuroscience and Medicine 4, INM-4, Forschungszentrum Jülich, 52428 Jülich, Germany

**Keywords:** functional connectivity, insula, non-tic symptoms, resting-state fMRI, salience network, tic disorder

## Abstract

Tic disorders (TD) are characterized by the presence of motor and/or vocal tics. Common neurophysiological frameworks suggest dysregulations of the cortico-striatal-thalamo-cortical (CSTC) brain circuit that controls movement execution. Besides common tics, there are other “non-tic” symptoms that are primarily related to sensory perception, sensorimotor integration, attention, and social cognition. The existence of these symptoms, the sensory tic triggers, and the modifying effect of attention and cognitive control mechanisms on tics may indicate the salience network's (SN) involvement in the neurophysiology of TD. Resting-state functional MRI measurements were performed in 26 participants with TD and 25 healthy controls (HC). The group differences in resting-state functional connectivity patterns were measured based on seed-to-voxel connectivity analyses. Compared to HC, patients with TD exhibited altered connectivity between the core regions of the SN (insula, anterior cingulate cortex, and temporoparietal junction) and sensory, associative, and motor-related cortices. Furthermore, connectivity changes were observed in relation to the severity of tics in the TD group. The SN, particularly the insula, is likely to be an important site of dysregulation in TD. Our results provide evidence for large-scale neural deviations in TD beyond the CSTC pathologies. These findings may be relevant for developing treatment targets.

## Significance Statement

Tic disorders (TD) are associated with a variety of symptoms beyond typical motor and vocal tics that affect sensory perception, attention, and social cognition. The presence of such non-tic symptoms suggests the potential involvement of the salience network (SN) in the pathophysiology of TD. While previous studies have predominantly focused on the cortico-striatal-thalamo-cortical circuitry, which is known to underlie tic generation and expression, we conducted resting-state functional magnetic resonance imaging to investigate the functional connectivity of the SN in TD. Notably, we observed impaired connectivity of the SN with relations to the tic symptom severity. Our research provided important evidence that the pathophysiology of TD involves the SN, which is highly relevant for developing treatment strategies.

## Introduction

Tic disorders (TD) are childhood-onset neurodevelopmental disorders characterized by a wide range of motor and/or vocal tics with various manifestations and severities (Hartmann and Worbe, 2018; Deeb et al., 2019; Pringsheim et al., 2019). Tics, defined as involuntary, repetitive movements and vocalizations (Leckman, 2003), can be quite debilitating, affecting various areas of a person's life (Müller-Vahl et al., 2010; Atkinson-Clement et al., 2022). In many cases, tics resolve on their own. In case they continue, a diagnosis of persistent motor or vocal TD or Tourette's syndrome may be applicable (DSM-5, American Psychiatric Association, 2013). TD are often associated with other psychiatric conditions such as obsessive–compulsive disorder (OCD), attention deficit hyperactivity disorder (ADHD), conduct disorder, and mood disorder (Conelea et al., 2011; Hirschtritt et al., 2015; Serajee and Mahbubul Huq, 2015). These comorbidities are an additional source of psychosocial distress and impairment in patients’ quality of life (Conelea et al., 2011; Smith et al., 2015; Evans et al., 2016).

Notably, there are also “non-tic” symptoms that cause debilities in TD. One of the important components of tics, the “premonitory urge”, often manifests as a non-motor, localized, or diffuse unpleasant sensation, such as the perception of pressure, hot or cold temperature, or tickling (Kwak et al., 2003; Reese et al., 2014). The correlation between tics and neuropsychological deficits is also notable in pediatric autoimmune neuropsychiatric disorders associated with group A beta-hemolytic streptococcus (PANDAS). These disorders manifest with the sudden onset of obsessive–compulsive symptoms, tics, or a combination of both, and their symptoms are heightened during group A streptococcal infections (Younger, 2023). Neuropsychological observations support a notion of TD that goes beyond a disorder of motor control and involves complex patterns of cognitive, emotional, and behavioral processes (Ruhrman et al., 2017), likely affecting the domains of sensory perception (Belluscio et al., 2011), sensory–motor integration (Friedrich et al., 2021), attention (Misirlisoy et al., 2015), and social cognition (Channon et al., 2004, 2012; Eddy and Cavanna, 2013a, 2015). Remarkably, these neuropsychological functions are closely related to salience-based information processing in the brain (Seeley, 2019; Lugrin et al., 2023). Dysfunctional alterations in the salience network (SN) have been observed in various psychiatric disorders, including ADHD, OCD, post-traumatic stress disorder, and schizophrenia (Berg et al., 2021; Huang et al., 2022; Jakobi et al., 2022; Tomiyama et al., 2022). In TD, the non-tic symptoms may rely on abnormalities of the cortical hubs within the SN, such as the anterior cingulate cortex (ACC), the ventral anterior insular cortex, and the temporoparietal junction (TPJ; Kucyi et al., 2012; Seeley, 2019; Uddin et al., 2019).

The SN acts as a neural hub that is primarily associated with detecting and prioritizing salient or relevant information in the environment and plays a vital role in determining the significance of sensory input (internally or externally) and guiding subsequent cognitive and motor responses (Schimmelpfennig et al., 2023). With these functions, it plays a critical role in orchestrating attention, guiding behavior, and regulating emotional responses (Menon and Uddin, 2010). The SN may be closely linked to the pathophysiology of TD. Individuals with TD often experience heightened sensory sensitivities and an inability to filter out irrelevant stimuli, leading to increased perceptions of tics and other sensory experiences. This heightened salience detection and atypical response within the network could contribute to the manifestation and exacerbation of tics and associated behavioral symptoms. The SN connectivity changes in TD have yet to be investigated. Currently, there is a lack of sufficient treatment strategies with respect to this disorder (Pringsheim et al., 2019; Chou et al., 2023; Johnson et al., 2023), indicating an urgent need for pathophysiological knowledge that can bridge the gap between behavior, the brain, and TD treatment.

Previous neuroimaging studies have focused mainly on dysregulations of the cortico-striatal-thalamo-cortical (CSTC) circuit as the core pathology in tic generation and execution (Albin and Mink, 2006; Ganos et al., 2013; Hartmann and Worbe, 2018). This circuit, comprising motor, affective, and limbic subsystems, plays a significant role in cognitive, emotional, and motor control (Bonelli et al., 2007; Orth et al., 2022). Changes within the CSTC circuits and cerebello-thalamo-cortical networks were also observed at the metabolic level in [^18^F]fluorodeoxyglucose (FDG) positron emission tomography (PET) imaging data. Studies using the [^18^F]FDG PET have shown that TD extends beyond the boundaries of basal ganglia circuits. This is evident in studies showing metabolic changes in regions outside the basal ganglia in TD (Pourfar et al., 2011; Buse et al., 2013). In particular, TD is characterized by significantly increased metabolic rates in the sensorimotor cortices, as demonstrated in several studies (Eidelberg et al., 1997; Stern et al., 2000; Lerner et al., 2007; Pourfar et al., 2011). To develop effective treatment strategies for TD, it is crucial to understand the widespread neurophysiological changes associated with the disorder beyond “tics”. Functional magnetic resonance imaging (fMRI) and resting-state fMRI, which detect temporally correlated large-scale networks in the brain such as the SN (Damoiseaux et al., 2006; Uddin et al., 2019; Huang et al., 2022), are the most effective methods for investigating these changes. Resting-state fMRI has the potential to improve clinical observation, diagnosis, and circuit-based interventions (Yang et al., 2020) and offers the advantage of providing a functional brain scan that is independent of the patient's cognitive ability, cooperation, or motivation.

Given the potential of the resting-state fMRI, we applied resting-state fMRI measures to examine if the connectivity of the cortical hubs of the SN showed abnormalities in TD and if these abnormalities might be associated with symptom severity. To that end, we conducted resting-state fMRI measurements in a group of TD patients and in a group of healthy controls (HC) and calculated the connectivity measures of the SN core regions. Using the Yale Global Tic Severity Scale (YGTSS), we investigated the relationships between brain and tic severity by correlating YGTSS scores with the connectivity measures of the SN regions. In addition, we examined the relationship between brain connectivity and the urge severity as measured by the Premonitory Urges for Tics Scale (PUTS). Based on the clinical evidence regarding the existence of non-tic presentations in TD, we hypothesized that patients with TD might show an abnormal connectivity between the SN core regions and the sensory and associative cortical areas.

## Materials and Methods

### Participants

Twenty-six adult patients [mean age: 33.0 years ± 10.6 (range, 19–58), seven females], who met the diagnostic criteria for a TD according to the ICD-10 (World Health Organization, 2010) and 25 matched HC [mean age: 32.3 years ± 11.8 (range, 18–59), six females] participated in the present study. Patients were recruited from the inpatient and outpatient psychiatric units at the University Hospital RWTH Aachen, Germany. Healthy volunteers were recruited through public advertisements and matched with respect to age and gender. We applied an independent sample *t* test and a *χ*^2^-test to examine the age and gender differences between the TD and HC groups. The analysis was performed using SPSS (IBM SPSS Statistics for Windows, Version 28.0 Armonk, NY: IBM). None of the HC participants met the criteria for a current or past psychiatric or neurological disease. For all participants, the exclusion criteria included current pregnancy, MRI contraindications, acute psychotic symptoms, severe head trauma, current substance use disorder, or a history of alcohol and substance abuse within the past 6 weeks. All participants were native German speakers and right-handed according to the Edinburgh Handedness Questionnaire. Of the 26 TD patients, 12 presented with a comorbid psychiatric diagnosis. This information was based on their medical records and confirmed by patients. Eleven patients received psychopharmacological treatment (for detailed diagnosis and medication list, see Table 1). The study was conducted at the University Hospital RWTH Aachen, Germany. The research protocol was approved by the local ethics committee (The Independent Ethics Committee, medical faculty, RWTH Aachen University, EK103-18). Human research in this study was conducted according to the principles expressed in the Declaration of Helsinki. All participants gave written informed consent to participate in the study and received financial compensation.

**Table 1.** Psychotropic medications and comorbidities in the TD group

	*n* (%)
Psychotropic medication	
No medication	12 (48)
Antipsychotics + SSRI	3 (12)
Antipsychotics + methylphenidate/atomoxetine	2 (8)
Antipsychotics	1 (4)
Antipsychotics + SSRI + THC	1 (4)
Tiapride	1 (4)
SSRI	1 (4)
SSRI + tiapride + methylphenidate/atomoxetine	1 (4)
THC	1 (4)
Methylphenidate/atomoxetine	1 (4)
Methylphenidate/atomoxetine + THC	1 (4)
Psychiatric comorbidities	
ADHD	4 (16)
OCD	3 (12)
ADHD + depression	1 (4)
OCD + ADHD	1 (4)
OCD + addiction (cannabis)	1 (4)
OCD + depression	1 (4)

### Clinical assessments

One day prior to the MRI measurement, the following assessments were conducted in the patient group:

1. YGTSS (Leckman et al., 1989; German version by H.-C. Steinhausen ). A clinician-rated scale, YGTSS is considered the gold standard for evaluating tics in patients with Tourette’s syndrome and other TD. This instrument allows assessment of the quantity, frequency, intensity, complexity, and interference of motor and vocal tics in the past week. Each domain is rated on a 6-point Likert scale (0, not at all; 5, very much), with a separate rating for “overall impairment” related to the patient's daily life and activities. The total motor tic score, the total vocal tic score, the total tic score, and the global tic severity score constitute the four summary scores.
2. PUTS (Woods et al., 2005), German version. PUTS is a self-rated scale for assessing urge severity in patients with tics.
3. Gilles de la Tourette Syndrome-Quality of Life Scale (GTS-QOL; Cavanna et al., 2008), German version . This self-rated scale measures the psychological, physical, obsessive–compulsive, and cognitive impact of Tourette’s syndrome and other TD.
4. Beck Depression Inventory-II (BDI-II; Beck et al., 1996), German version (Kühner et al., 2007). BDI-II is a questionnaire that measures the severity of depressive symptoms.
5. Yale–Brown Obsessive–Compulsive Scale (Y–BOCS; Goodman et al., 1989), German version (Hand and Büttner-Westphal, 1991). This clinician-rated scale facilitates evaluations of OCD-related symptoms.

### MR imaging protocol

Imaging data were obtained from a 3.0 T Siemens Prisma fit MRI scanner (Magnetom, Siemens Medical Systems) with a 32-channel head coil. A resting-state fMRI measurement was conducted for all participants. Participants were instructed to lie still with their eyes open, not fall asleep and let their thoughts run free during the resting-state measurement. All participants confirmed that they had followed these directions. The whole-brain fMRI measurement was performed with an echo planar imaging sequence [repetition time (TR) = 2,000 ms; echo time (TE) = 28 ms; flip angle = 77°; voxel size = 3 × 3 mm; matrix size = 64 × 64 × 64; 34 transverse slices (interleaved acquisition); and 210 images]. Structural images were acquired using a T1-weighted magnetization prepared rapid acquisition with gradient echo sequence (TR = 2,000 ms; TE = 30.3 ms; inversion time = 900 ms; flip angle = 9°; voxel size = 1 × 1 mm; 176 sagittal slices; 1 mm slice thickness; field of view = 256 × 256 mm^2^; GRAPPA factor 2).

### MRI data analysis

#### Preprocessing

Both the anatomical and functional image analyses were conducted using the MATLAB-based CONN toolbox (version 22.a; Whitfield-Gabrieli and Nieto-Castañón, 2012; Nieto-Castanon and Whitfield-Gabrieli, 2022) implemented in SPM12 (Penny et al., 2011). To avoid T1 saturation effects, the first five images were excluded from the analysis.

Functional and anatomical data were preprocessed using a flexible preprocessing pipeline (Nieto-Castanon, 2020) including realignment with correction of susceptibility distortion interactions, slice-timing correction (STC), outlier detection, direct segmentation and MNI space normalization, and smoothing. Functional data were realigned using SPM realign and unwarp procedure (Andersson et al., 2001), where all scans were coregistered to a reference image (first scan of the first session) using a least squares approach and a six-parameter (rigid body) transformation (Friston et al., 1995) and resampled using b-spline interpolation to correct for motion and magnetic susceptibility interactions. Temporal misalignment between different slices of the functional data (acquired in interleaved Siemens order) was corrected following SPM STC procedure (Henson et al., 1999; Sladky et al., 2011), using sinc temporal interpolation to resample each slice BOLD time series to a common midacquisition time. Potential outlier scans were identified using artifact detection tools (Whitfield-Gabrieli et al., 2011) as acquisitions with framewise displacement above 0.9 mm or global BOLD signal changes above five standard deviations (Nieto-Castanon, n.d.; Power et al., 2014), and a reference BOLD image was computed for each subject by averaging all scans excluding outliers. Functional and anatomical data were normalized into the standard MNI space; segmented into gray matter, white matter, and CSF tissue classes; and resampled to 2 mm isotropic voxels following a direct normalization procedure (Nieto-Castanon, n.d.; Calhoun et al., 2017) using the SPM unified segmentation and normalization algorithm (Ashburner and Friston, 2005; Ashburner, 2007) with the default IXI-549 tissue probability map template. Last, functional data were smoothed using spatial convolution with a Gaussian kernel of 8 mm full-width at half-maximum.

In addition, functional data were denoised using a standard denoising pipeline (Nieto-Castanon, 2020) including the regression of potential confounding effects characterized by white matter time series [five component-based noise correction method (CompCor) noise components], CSF time series (five CompCor noise components), motion parameters and their first-order derivatives (12 factors; Friston et al., 1996), outlier scans (below 82 factors), session effects and their first-order derivatives (two factors), and linear trends (two factors) within each functional run, followed by bandpass frequency filtering of the BOLD time series (Hallquist et al., 2013) between 0.008 and 0.09 Hz. CompCor (Behzadi et al., 2007; Chai et al., 2012) noise components within the white matter and CSF were estimated by computing the average BOLD signal as well as the largest principal components orthogonal to the BOLD average, motion parameters, and outlier scans within each subject's eroded segmentation masks. From the number of noise terms included in this denoising strategy, the effective degrees of freedom of the BOLD signal after denoising were estimated to range from 43.3 to 70.2 (average 67.9) across all subjects (Nieto-Castanon, n.d.).

#### First- and second-level analyses

Seed-based connectivity (SBC) maps were estimated characterizing the patterns of functional connectivity with high-performance computing-independent component analysis (ICA) networks (Nieto-Castanon and Whitfield-Gabrieli, 2022). The functional connectivity strength was represented by Fisher-transformed bivariate correlation coefficients from a weighted general linear model (GLM; Nieto-Castanon, 2020), defined separately for each pair of seed and target areas, modeling the association between their BOLD signal time series. In order to compensate for possible transient magnetization effects at the beginning of each run, individual scans were weighted by a step function convolved with an SPM canonical hemodynamic response function and rectified.

Group-level analyses were performed using a GLM (Nieto-Castanon, 2020). For each individual voxel, a separate GLM was estimated, with first-level connectivity measures at this voxel as dependent variables (one independent sample per subject and one measurement for the rest condition) and groups as independent variables. Voxel-level hypotheses were evaluated using multivariate parametric statistics with random effects across subjects and sample covariance estimation across multiple measurements. Inferences were performed at the level of individual clusters (groups of contiguous voxels). Cluster-level inferences were based on parametric statistics from the Gaussian random field theory (Worsley et al., 1996; Nieto-Castanon, 2020). Results were thresholded using a combination of a cluster-forming *p* < 0.001 voxel-level threshold and a false discovery rate (FDR)-corrected p_FDR _< 0.05 cluster-size threshold (Chumbley et al., 2010). Additionally, linear regressions were performed between the YGTSS score (tic severity) and brain connectivity of the seed regions as well as the PUTS score (urge severity) and brain connectivity. The correction of these analyses had the same threshold (cluster-level p_FDR _< 0.05, voxel level *p* < 0.001, uncorrected).

#### Quality assurance

All structural and functional MRI scans were visually inspected to ensure that participants had no significant brain atrophy. After preprocessing, the quality of the preprocessed data was inspected using the quality assurance (QA) plots available in the CONN toolbox (QA normalization, registration, and motion). Due to excessive motion (continuous head movements, not specific to tics), one patient was removed from further analysis resulting in *n* = 25 patients and *n* = 25 HC. For this patient, >50% of the scans were detected as outlier scans.

## Results

### Sample characteristics

Patient and HC groups did not differ in age [*t*_(48)_ = −0.09; *p* = 0.928] or gender (*χ*^2^ = 0; *p* = 1.0). The patients had a mean YGTSS total tic score of 22.9 ± 8.2 (range, 10–42) and a GTS-QOL score of 28.1 ± 18.5 (range, 1–69). The average PUTS score was 24.9 ± 5.7 (range, 10–33) with 11 patients having a medium intensity (range, 12.5–24.5), eight patients a high intensity (range, 25–30.5), and five patients an extremely high intensity (≤31) of premonitory urges for tics. The average BDI-II score was 12.0 ± 11.0 (range, 0–43) with five patients reporting moderate to severe depressive symptoms (range, 20–63) and the remaining minimal to mild depressive symptoms (≤19). Out of 25 patients, 11 described obsessive–compulsive symptoms resulting in an average Y–BOCS score of 6.7 ± 10.2 (range, 0–37), with four patients having moderate symptoms (range, 16–23) and one patient having extreme symptoms (range, 32–40), while the rest reported subclinical to mild symptoms (≤15; Table 2).

**Table 2.** Clinical scores of TD patients (*n* = 25)

	Mean	SD	Min	Max
YGTSS	22.9	8.2	10	42
GTS-QOL	28.1	18.5	1	69
PUTS	24.9	5.7	10	33
BDI-II	12.0	11.0	0	43
Y–BOCS	6.7	10.2	0	37

BDI-II, Beck Depression Inventory-II; GTS-QOL, Gilles de la Tourette Syndrome-Quality of Life Scale; PUTS, Premonitory Urges for Tics Scale; Y–BOCS, Yale–Brown Obsessive–Compulsive Scale; YGTSS, Yale Global Tic Severity Scale.

### Resting-state fMRI: SBC

**Figure 1.**
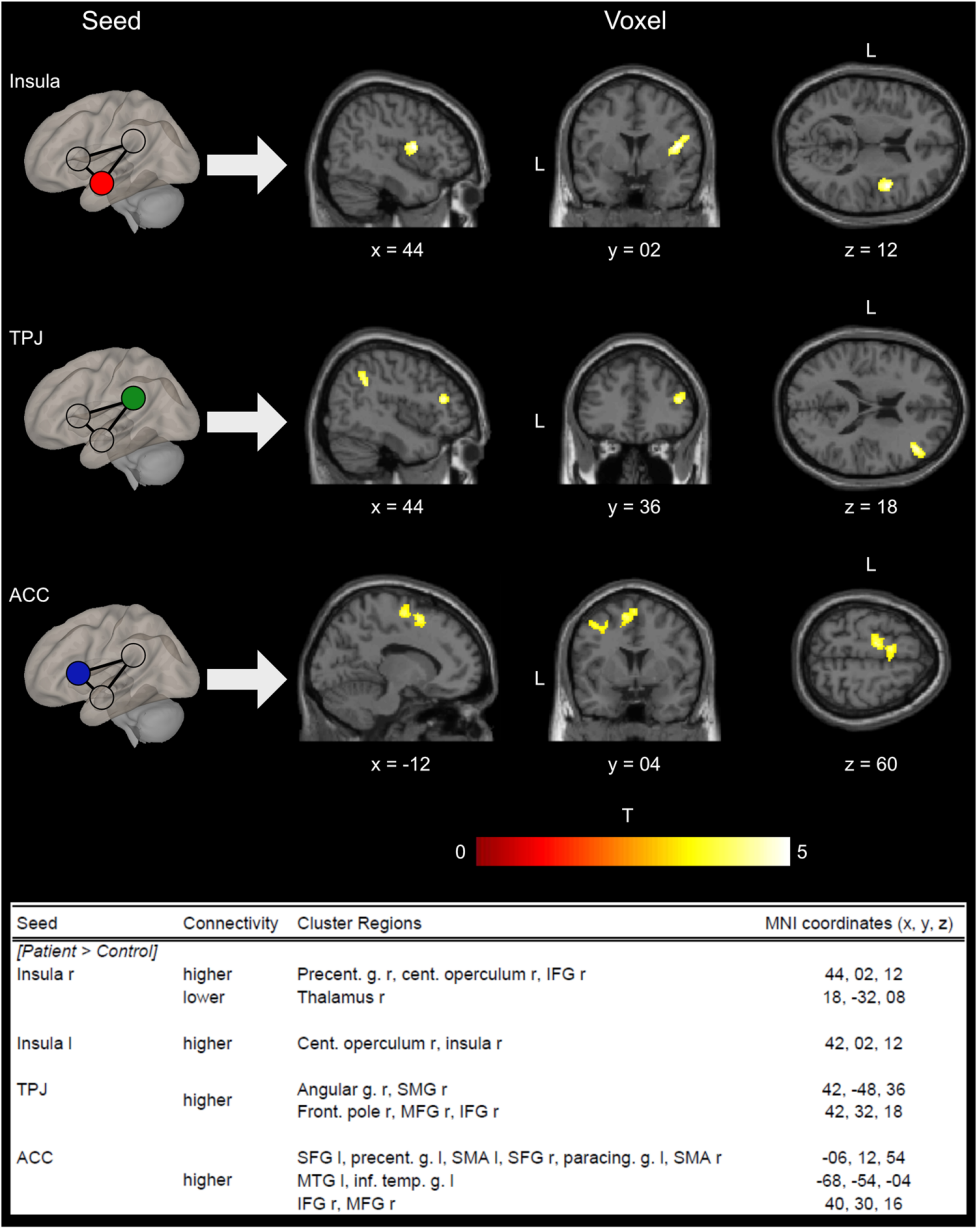
SBC results for the seeds’ right insula (higher connectivity), bilateral TPJ, and the ACC and the contrast (patient > control). The red–yellow–white color indicates increased connectivity. The table lists the individual clusters of each seed region in detail with the corresponding MNI coordinates. ACC, anterior cingulate cortex; TPJ, temporoparietal junction; angular g., angular gyrus; cent. operculum, central operculum; front. pole, frontal pole; IFG, inferior frontal gyrus; inf. temp. g., inferior temporal gyrus; MFG, middle frontal gyrus; MTG, middle temporal gyrus; paracing. g., paracingulate gyrus; precent. g., precentral gyrus; SMA, supplementary motor area; SMG, supramarginal gyrus.

The differences in functional connectivity between the patient and control groups, and the correlation of connectivity measures with tic symptom severity measured by YGTSS and PUTS in the patient group, were examined by means of SBC analyses. For the between-group connectivity analysis, the major nodes of the SN were chosen as the seed regions. Compared to their HC counterparts, patients with TD showed significantly different connectivity in the right and left insula, the TPJ, and the ACC (Fig. 1). 

The right insula showed significantly higher functional connectivity to the right precentral gyrus, the right central operculum, and the right inferior frontal gyrus (IFG) as well as lower connectivity to the right thalamus. The left insula showed significantly higher connectivity to the central operculum and the right insula.

As a seed region, the TJP revealed higher connectivity to clusters including the right frontal pole, the right middle frontal gyrus (MFG), and the IFG as well as the right angular gyrus and the right supramarginal gyrus in the patient group compared with the HC group.

The ACC seed showed significantly higher connectivity to the bilateral superior frontal gyrus (SFG), the bilateral supplementary motor area (SMA), the left paracingulate gyrus, the left precentral gyrus, the right IFG, and the right MFG as well as the left middle temporal gyrus and the left inferior temporal gyrus.

**Figure 2.**
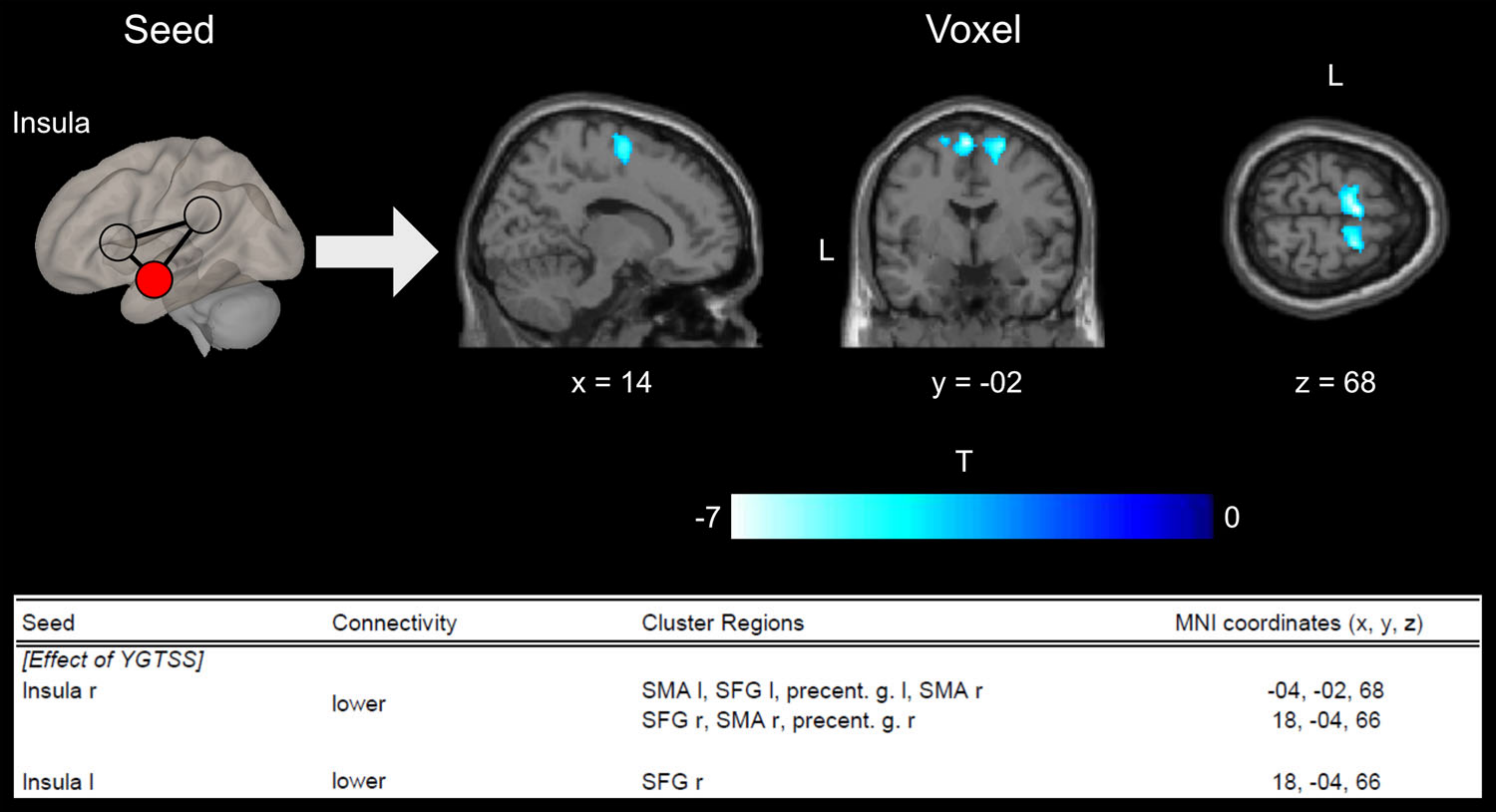
SBC results for the right insula seed in correlation to the YGTSS score. The blue–white coloring indicates decreased connectivity. The table indicates the cluster of the right and left insula seed with the corresponding MNI coordinates. Precent. g., precentral gyrus; SFG, superior frontal gyrus; SMA, supplementary motor area.

To investigate the correlation between patients’ clinical scores and their connectivity, we conducted an additional analysis, which revealed a negative correlation between the connectivity of the left insula and the right SFG and the tic severity as measured by the YGTSS (Fig. 2). We additionally revealed a negative correlation between the connectivity of the right insula and clusters including the bilateral SFG, the bilateral SMA, and the bilateral precentral gyrus and the tic severity as measured by the YGTSS. The other regions of the SN did not reveal significant correlations between the patients’ clinical scores and their connectivity. No significant correlation was found between the PUTS score and brain connectivity of any SN region.

## Discussion

The existence of “non-tic” symptoms in TD and the prevalence of other psychiatric comorbidities suggest that TD is not simply a movement disorder, but a complex disorder involving wide-ranging brain pathologies that may occur beyond the motor-related brain areas. The present study provides evidence that SN connectivity is altered in patients with TD. While most previous studies investigating altered connectivity in TD had focused on the neural networks underlying tic generation and expression, and indicated the pathological deviations within the CSTC circuit, we used resting-state brain scans to explore the functional connectivity of the SN in this disorder. The seed-to-voxel functional connectivity analyses of the SN cortical nodes (insula, ACC, and TPJ) revealed increased connectivity to the sensory, associative, and motor-related cortices. The insula was found to play a salient role in a disturbed connectivity to the frontal lobe in correlation with the tic severity. By exploring the altered SN connectivity, which may have implications in non-tic symptoms, our study adds to the understanding of large-scale brain network involvement in TD. Resting-state fMRI may be an appropriate brain mapping method to evaluate regional interactions, which occur within the large-scale brain networks beyond the motor-related areas and are not limited to temporary tics. Previous resting-state studies have already revealed an involvement of the default mode network (DMN) and the frontoparietal network in the TD pathophysiology (Fan et al., 2018; Ramkiran et al., 2019; Zito et al., 2021). ICAs have shown a significant increase in the coupling between and within the amygdalae and increased integration of the dorsomedial prefrontal cortex (PFC) in the DMN connectivity and decreased integration of the inferior parietal cortex in the frontoparietal network connectivity (Werner et al., 2010; Fan et al., 2018). Studies using graph theoretical measures have found increased basal ganglia–cortical and thalamo-cortical connectivity, reduced cortico-cerebellar connectivity, and increased connectivity of the dorsal insula with frontostriatal nodes in patients with TD compared with controls (Worbe et al., 2012; Tinaz et al., 2015; Ramkiran et al., 2019). SBC analyses in TD have revealed greater connectivity between the temporal gyri, the insula, and the putamen and between the orbitofrontal cortex and the ACC as well as between the sensory motor cortex and the insula (Tinaz et al., 2014; Bhikram et al., 2020). Another study has identified TD through multivariate analyses based on a support vector machine using resting-state data from a network including the striatum, the frontoparietal cortical areas, and the cerebellum (Zito et al., 2021). Our observations add to previous findings pertaining to altered functional connectivity in TD.

Generally, it seems that the notion of non-tic symptoms has not been sufficiently addressed in the common neurophysiological models of TD, which have focused mainly on CSTC pathologies (Szejko, 2022). However, sensorimotor integration, a process through which the central nervous system integrates sensory information to plan motor responses, may be crucial for the performance of efficient movements (Nijs et al., 2012; Seki and Fetz, 2012). Evidence suggests that the basal ganglia are involved in the inhibition of certain sensory inputs, which, in combination with faulty sensorimotor cortex projections, can lead to disturbances in movement control. Individuals with TD may have impairments in these functions (Abbruzzese and Berardelli, 2003), likely affecting sensorimotor integration and, consequently, motor control (Patel et al., 2014). The increased binding of stimulus- and response-related cues in visual processes and the altered sensorimotor integration processes in TD suggest an increased perceptual and action binding in the somatosensory area (Nowak et al., 2005; Friedrich et al., 2021). In this context, the integration of perceptions into processes pertaining to planning, execution, and adaptation of complex movements has been shown to be impaired in TD (Kim et al., 2019). The ability to at least partially control tics suggests a link to motor learning with tighter stimulus–response binding (Ganos et al., 2014; Brandt et al., 2016a). Remarkably, TD is associated with other non-tic symptoms with higher social relevance, including altered social cognition, social disinhibition, and a phenomenon called non-obscene socially inappropriate symptoms (NOSIS), which is described as an urge to perform behaviors that are socially disruptive or offensive to others (Kurlan et al., 1996; Eddy and Cavanna, 2013b). While no explanatory framework is available regarding NOSIS, the combination of excessive deliberation about other people's mental states and an inability to inhibit impulses may explain why some people with TD experience NOSIS (Eddy and Cavanna, 2013a,b). According to Gilles de la Tourette, tics may be triggered by mirroring the movements or vocalizations of others, known as echopraxia or echolalia (Ganos et al., 2012; Brandt et al., 2016b). Also, some of the complex tics, such as coprolalia, swearing tics, and the urge to imitate other people's speech and behavior, are tightly bound to social interactions (Kurlan et al., 1996; Eddy and Cavanna, 2013b; Ganos et al., 2016). Increased salience in TD may augment an heightened awareness of external stimuli, which, along with impulse dysregulations, may contribute to these special tics (Eddy and Cavanna, 2015). Our observation of SN involvement is in line with the salience processing that pertains to the socially inappropriate drives, echophenomena, and NOSIS.

The SN performs a pivotal role in detecting salient stimuli from a continuous flow of sensory information acting on the senses. According to Menon and Uddin (2010), the SN is responsible for initiating control signals to regulate behavior and the homeostatic state. The SN, which serves as a switch between the central executive network and the DMN, may be impaired in TD, leading to insufficient salience detection and filtering information. Consequently, individuals with TD may often experience hypersensitivity to external stimuli, as well as a tendency to overthink and hypermentalize (Belluscio et al., 2011; Isaacs and Riordan, 2020).

The insula is a major node of the SN, and its role in TD has already been pointed out in numerous studies, particularly in association with the generation of premonitory sensations or “urges” that commonly precede tics (Tinaz et al., 2015; Draper et al., 2016; Conceição et al., 2017; Jackson et al., 2020). In fact, electrical stimulation of the insular cortex and the parietal operculum elicits unpleasant somatosensory or visceral sensations (Leckman and Riddle, 2000). The presence of these premonitory urges has been shown to be associated with tic severity in TD (Kyriazi et al., 2019). The premonitory sensations are usually attenuated or canceled by the execution of the tics (Leckman, 2002; Brandt et al., 2016a). Thus, there may be a strong interaction between perceptual and movement processes, which is underscored by our findings of increased connectivity between the insula and the motor areas. Previous findings have indicated an increased connectivity between the anterior insula and the frontostriatal areas in TD patients compared with controls (Tinaz et al., 2015). The functional connectivity between the right dorsal anterior insula and the left dorsomedial PFC has been found to correlate positively with the urge severity, indicating that sensory cortices as well as the limbic and paralimbic areas, such as the ACC, the insula, and the amygdala, may be involved in the generation of premonitory urges (Tinaz et al., 2015). We also observed an increased connectivity between the right insula and the central operculum involved in sensory and cognitive processing (Mălîia et al., 2018) as well as the precentral gyrus, involved in sensorimotor integration (Cooke and Graziano, 2004). The involvement of sensory processing has already been highlighted in TD, reflecting a hypersensitivity to the external stimuli (Cohen and Leckman, 1992; Belluscio et al., 2011; Buse et al., 2015). In fact, tics have been reported to be exacerbated in response to various visual, auditory, and tactile stimuli (Commander et al., 1991; Eapen et al., 1994; Janik et al., 2018; Isaacs and Riordan, 2020). Our results are consistent with the findings by Bhikram et al. (2020), who have reported an increased connectivity between the insula and the temporal gyri, which may reflect the insula's role in increasing the salience of premonitory urges. Data from [^18^F]FDG PET imaging studies similarly reported increased brain activity in the insula and the superior temporal gyrus (Stern et al., 2000).

An increased connectivity between the TPJ and the associative areas in the parietal and frontal lobe, and areas involved in visuospatial perception, i.e., the angular gyrus, has been revealed in our data. The TPJ as part of the SN is involved in a variety of processes, including sensorimotor integration (Blanke and Mohr, 2005), imitation processes (Spengler et al., 2010), social cognition, and stimulus-driven attentional functions (Corbetta et al., 2008). In line with our observations, TD has been found to be associated with increased activation of the TPJ using fMRI and [^18^F]FDG PET, with alterations of the TPJ activity having been correlated with coprolalia, echophenomena, and NOSIS (Stern et al., 2000; Eddy et al., 2016).

In the current study, the evaluation of the connectivity patterns of the ACC as part of the SN revealed an increased connectivity between the SN and motor areas, i.e., the SMA and the SFG, and areas involved in visuospatial perception, i.e., the temporal pole and the middle temporal gyrus. The ACC also showed a higher connectivity to the PFC, which is involved in attention control. Pathologies of attention rely on the SN (Menon and Uddin, 2010) and may interact with the tics (Misirlisoy et al., 2015). Previous findings suggest that individuals with TD are more likely to experience attention deficits compared with individuals without TD, particularly in tasks involving cognitive flexibility, divided attention, and response inhibition (Johannes et al., 2001; Kurvits et al., 2020). Additionally, males with TD have shown altered reactions toward predictable versus unpredictable stimuli in the brain regions that play a significant role in attention control, likely indicating an altered allocation of attention toward those stimuli (Buse et al., 2017). Generally, attention seems to have modulating effects on tics: while focusing attention on the tics intensifies them significantly (Misirlisoy et al., 2015; Herrmann et al., 2019), the frequency of tics decreases when individuals focus on visual stimuli (Brandt et al., 2015) or direct their attention to a motor task (Misirlisoy et al., 2015; Stiede and Woods, 2020). ACC dysfunction has been linked to mild cognitive deficits in attention and inhibition in TD (O’Neill et al., 2019), which may be reflected by the increased connectivity between the ACC and the frontal regions. The results can additionally be strengthened by available [^18^F]FDG PET studies that revealed significant increases in the metabolic activity of the ACC and frontal cortices associated with attentional and visuospatial dysfunction as well as coprolalia and echophenomena (Braun et al., 1995; Jeffries et al., 2002).

We explored the connectivity of the insula in greater depth through the correlations of tic severity as measured by YGTSS. Tic severity has already been shown to be correlated with the engagement of the SMA, the precentral gyrus, and the MFG across different tasks (Polyanska et al., 2017) and with increased connectivity between the putamen and the sensorimotor cortex (Bhikram et al., 2020). In the current analysis, we observed the changes that occurred in the insular functional connectivity in correlation with tic severity. A lower functional connectivity between the insula and the areas within the SFG was observed in patients with higher tic severity. The SFG is involved in self-awareness and impulse control and modulates inhibitory control and motor urgency (Hu et al., 2016). The reduced functional connectivity between the insula and the SFG at higher tic severity may explain the lower inhibitory control in severely affected patients, likely establishing a link between the altered processing of salient information and decreased inhibitory control in TD.

It is generally acknowledged that, in addition to the typical motor-related tics, patients with TD have a wide range of symptoms that primarily affect social cognition, attention, and sensory perception. The SN, and particularly the insula, is critical for the detection of internal and external stimuli and for the coordination of the brain's neural resources in response to those stimuli. The altered connectivity between the SN and the motor, sensory, and attentional regions suggests that SN pathologies likely trigger the mechanisms that underlie the tic and non-tic symptoms in TD.

While this study aimed to shed light on the deviations of the SN connectivities in TD, it is essential to acknowledge several limitations that should be considered when interpreting the results. One of the primary limitations of this investigation is the limited sample size of the study, which has implications for the robustness and applicability of the results to the broader TD population. Furthermore, the small sample size may have challenged detecting subtle but potentially important differences in the SN between TD patients and HC. As a result, the statistical power of the study may be compromised, increasing the risk of both Type I and Type II errors. Generally, the limited sample size makes it difficult to explore potential moderating factors or sources of heterogeneity within the TD group, such as age of onset, tic severity, comorbid conditions, and medication status. These factors could significantly influence the functional connectivity patterns in the SN and contribute to the variability within the TD population. The possibility of psychiatric medications having their effects through network reorganization in the brain cannot be ruled out (Schulz and Steimer, 2000). Many patients with TD may show additional conditions of Axis I disorders (Hirschtritt et al., 2015), as was the case with our sample. The presence of various comorbid disorders increased the heterogeneity of our sample, thus limiting the specificity of our results.

Furthermore, a threshold of 0.5 mm for frame displacement in fMRI motion scrubbing was applied in preprocessing of the data in the current study to strike a balance between mitigating motion artifacts and minimizing the risk of substantial data loss. While this threshold is commonly used in fMRI studies, the choice of optimal threshold should depend on specific factors such as the characteristics of the study population. Motion is a significant concern in TD; therefore, studies of this population might opt for more stringent thresholds to minimize any potential influence of motion. We suggest contemplating the use of conservative fMRI motion correction strategies in large sample studies of TD.

## Conclusion

The above limitations notwithstanding, the results of our study add to the current understanding of the widespread dysfunction of large-scale brain networks in TD. Most specifically, we have successfully demonstrated an imbalanced connectivity between the main hubs of the SN and the associative and sensory processing areas in TD. The reduced connectivity of the insula and the SFG has been found to correlate with the TD symptom severity, highlighting a link between salience processing and inhibitory control. These findings may afford clinically relevant insights into the neurophysiology of TD beyond the CTSC circuits, providing a framework for understanding the underlying neurophysiology of the sensory tic triggers and the non-tic symptoms. This knowledge can potentially lead to the identification of new therapeutic modalities in future research pertaining to the sensory triggers of tics.

## Synthesis

Reviewing Editor: Christopher Colwell, UCLA School of Medicine Decisions are customarily a result of the Reviewing Editor and the peer reviewers coming together and discussing their recommendations until a consensus is reached. When revisions are invited, a fact-based synthesis statement explaining their decision and outlining what is needed to prepare a revision will be listed below. The following reviewer(s) agreed to reveal their identity: David Young.

The current manuscript presents a study of functional connectivity (FC) MRI in adults with tic disorders (TD). The manuscript reports increased FC between multiple selected brain regions in TS compared to a sample of healthy controls (HC), as well as correlations between FC and tic severity. Studying TD in humans with FC is commendable and necessary, as there are not many investigators leveraging these tools in the TD world. However, the literature is fraught with low sample studies and results that are unlikely to be replicated. There are two major methodological issues here.

One major methodological issue to be addressed carefully is head motion. Since head motion systematically affects FC correlations, it very well could lead to a finding of increased FC in a movement disorder group compared to controls, as reported in this manuscript. Importantly, head motion can be an issue even when metrics of mean motion are not significantly different between the groups (see Satterthwaite et al. 2012 NeuroImage, Van Dijk et al. 2012 NeuroImage, Power et al. 2012 NeuroImage, Power et al. 2014 NeuroImage). Fortunately, the discovery of the motion problem has led many groups to develop methods that aim to mitigate its affects, and systematic comparisons have been carried out to determine which methods are best for certain types of studies and research questions. The most thorough evaluation demonstrated that global signal regression + scrubbing (frame censoring, spike regression) best mitigates motion artifact when the questions of interest involve comparing groups (Ciric et al. 2017 Neuroimage; Satterthwaite et al. 2017 Human Brain Mapping). In the present manuscript, the description of preprocessing methods is very brief. For example, "outlier detection (ART-based identification of outlier scans for scrubbing)" is not sufficient in detail. Was scrubbing used? What were the parameters and thresholds? How was movement measured? What is considered an "outlier," and is "outlier detection" the same or different from scrubbing? Without these details, it is very difficult to evaluate the soundness of the methods, and we am left thinking that motion is entirely driving the result. Since motion is an issue in this population and this study compares groups, testing for motion artifact and strong justification for the choices employed are needed.

The second major issue is small sample size with 20 participants per group. Given that true effect sizes are small, extremely large samples (1000's of subjects) are needed for reliable brain-behavior correlations (Marek et al. 2022 Nature). Even rebuttal papers arguing for smaller samples show that at a minimum 100's are still needed. Therefore, with only 20 subjects, it seems highly unlikely that the correlations between FC and YGTSS are reliable.

Still we recognize that it is difficult to collect resting state fMRI from patient populations. There are at least two ways to deal with this. Most appealing would be to set up a connection with collaborators who also have resting state fMRI data in patients with TS. That way, the authors can bolster the sample and test for replicability. Alternatively, the limitations section can be expanded. There are analytic approaches that can be implemented to test for reliability and generalizability of the results. Without these steps, there is the concern that the manuscript does not help us get to a better mechanistic understanding of TS, but rather adds to the confusion and replication issues.

Other comments:

The Introduction and Discussion sections could be better focused on introducing the concept of the SN and its relevance to TS notably in conjunction with the CSTC system.

The Results would be strengthened with available FDG PET MRI fused imaging.

Please consider citing new article: Younger DS. Pediatric neuropsychiatric disorders with motor and nonmotor phenomena. Handb Clin Neurol. 2023;196:367-387).
